# The Impact of Body Mass Index on In-Hospital Mortality in Post-Cardiac-Arrest Patients—Does Sex Matter?

**DOI:** 10.3390/nu15153462

**Published:** 2023-08-04

**Authors:** Michał Czapla, Adrian Kwaśny, Małgorzata Słoma-Krześlak, Raúl Juárez-Vela, Piotr Karniej, Sara Janczak, Aleksander Mickiewicz, Bartosz Uchmanowicz, Stanisław Zieliński, Marzena Zielińska

**Affiliations:** 1 Department of Emergency Medical Service, Wrocław Medical University, 51-616 Wrocław, Poland; michal.czapla@umw.edu.pl (M.C.); aleksander.mickiewicz@umw.edu.pl (A.M.); 2 Group of Research in Care (GRUPAC), Faculty of Health Sciences, University of La Rioja, 26006 Logroño, Spain; raul.juarez@unirioja.es (R.J.-V.); piotr.karniej@wsb.wroclaw.pl (P.K.); 3 Institute of Dietetics, Academy of Business and Health Science, 90-361 Łódź, Poland; a.kwasny@wsbinoz.pl; 4 Department of Human Nutrition, Department of Dietetics, Faculty of Health Sciences in Bytom, Medical University of Silesia in Katowice, 40-055 Katowice, Poland; msloma-krzeslak@sum.edu.pl; 5 Faculty of Finances and Management, WSB MERITO, University in Wroclaw, 53-609 Wrocław, Poland; 6 Student Research Group, Department of Vascular, General and Transplantation Surgery, Wrocław Medical University, 50-556 Wrocław, Poland; sara.janczak@student.umw.edu.pl; 7 Department of Nursing and Obstetrics, Faculty of Health Sciences, Wroclaw Medical University, 51-618 Wrocław, Poland; 8 Department and Clinic of Anaesthesiology and Intensive Therapy, Faculty of Medicine, Wrocław Medical University, 50-556 Wrocław, Poland; stanislaw.zielinski@umw.edu.pl (S.Z.); marzena.zielinska@umw.edu.pl (M.Z.)

**Keywords:** BMI, body mass index, cardiac arrest, sex differences

## Abstract

Background: A number of factors influence mortality in post-cardiac-arrest (CA) patients, nutritional status being one of them. The aim of this study was to assess whether there are sex differences in the prognostic impact of BMI, as calculated on admission to an intensive care unit, on in-hospital mortality in sudden cardiac arrest (SCA) survivors. Methods: We carried out a retrospective analysis of data of 129 post-cardiac-arrest patients with return of spontaneous circulation (ROSC) admitted to the Intensive Care Unit (ICU) of the University Teaching Hospital in Wrocław between 2017 and 2022. Results: Female patients were significantly older than male patients (68.62 ± 14.77 vs. 62.7 ± 13.95). The results of univariable logistic regression analysis showed that BMI was not associated with the odds of in-hospital death in either male or female patients. In an age-adjusted model, age was an independent predictor of the odds of in-hospital death only in male patients (OR = 1.034). In our final multiple logistic regression model, adjusted for the remaining variables, none of the traits analysed were a significant independent predictor of the odds of in-hospital death in female patients, whereas an initial rhythm of ventricular fibrillation or pulseless ventricular tachycardia (VF/pVT) was an independent predictor of the odds of in-hospital death in male patients (OR = 0.247). Conclusions: BMI on admission to ICU is not a predictor of the odds of in-hospital death in either male or female SCA survivors.

## 1. Introduction

In-hospital cardiac arrests (IHCA) and out-of-hospital cardiac arrests (OHCA) are a global public health problem. It is estimated that the annual incidence of OHCA in Europe ranges between 55 and 113 per 100,000 inhabitants, depending on the country [1,2,3]. Return of spontaneous circulation (ROSC) is achieved on-site following the administration of advanced life support (ALS) in only 10–50% of OHCA patients [4]. However, fewer than 23% of patients survive to hospital admission, and 10% survive to hospital discharge [5]. Other public health challenges and problems include obesity and overweight, which affect more than 53% of the European Union inhabitants [6]. It is well known that obesity and overweight is a factor that worsens prognosis in CA survivors [7]. Body mass index (BMI) is a simple, basic tool used by medical personnel to estimate the amount of body fat [8].

When a person suffers a CA, it is necessary to start cardiopulmonary resuscitation (CPR) as quickly as possible. According to the European Resuscitation Council Guidelines, chest compressions in adult patients in CA should be 5–6 cm deep [9]. However, this chest compression depth might be insufficient in patients with obesity due to changes in their anatomy [10,11]. A number of studies have found a correlation between high BMI and diabetes (DM), hypertension (HT) and serious heart problems, such as heart failure (HF) and arrhythmia [12,13,14]. All these conditions increase the risk of OHCA and IHCA and reduce the odds of ROSC [15]. Studies have also shown that malnourished patients have a significantly higher risk of suffering a CA and worse outcomes [16].

Studies analysing sex differences in treatment outcomes of post-CA patients have found that female patients have more unfavourable prognostic factors. They tend to be older and are less likely to suffer a CA in a public location or have an initial shockable rhythm [17,18]. While there have been several studies assessing the impact of BMI on prognosis in OHCA and IHCA survivors [15,19,20], very few of them have analysed sex differences in this regard. In our previous study (in which we did not disaggregate patients by gender), BMI results were not a factor which, on their own (unconditionally), altered the odds of mortality, but the risk of in-hospital mortality (expressed as hazard ratio—the risk over the study time period) increased with an increase in BMI in patients admitted to the intensive care unit (ICU) after IHCA and OHCA [21]. Considering the lack of sources from the literature and the results of our own research, we decided to investigate gender differences in this aspect.

The aim of this study was to assess whether there are sex differences in the prognostic impact of BMI, as calculated on admission to ICU, on in-hospital mortality in sudden cardiac arrest survivors.

## 2. Materials and Methods

### 2.1. Study Design and Setting

We carried out a retrospective analysis of data of 129 patients admitted to the Intensive Care Unit (ICU) of the University Teaching Hospital in Wrocław between 2017 and 2022, who had survived an in-hospital or out-of-hospital cardiac arrest (ICD10: I46). The study has been reported following the STROBE (Strengthening the Reporting of Observational Studies in Epidemiology) guidelines.

### 2.2. Study Population and Data

The study included all patients who met the following criteria: age ≥18 years, admission to ICU for SCA, and BMI on admission entered in medical records. The study excluded patients who had suffered a SCA following an injury or suicide attempt. Ultimately, 129 patients were included in the study. We analysed the following patient data: age, BMI, length of hospital stay, initial rhythm of CA, comorbidities (chronic kidney disease (CKD), heart failure (HF), diabetes (DM), hypertension (HT), prior cerebral stroke (CS) and acute coronary syndrome (ACS)) and laboratory test results: albumen, sodium (Na), potassium (K), procalcitonin (PCT) and high-sensitivity *C*-reactive protein (hsCRP). BMI results were interpreted/assessed in accordance with the WHO criteria: normal weight (BMI 18.5–24.9), pre-obese (BMI 25–29.9) and obese (BMI ≥ 30). As an alternative, patients were divided into obese (BMI ≥ 30) and non-obese (BMI < 30) groups. Each patient’s BMI was calculated and updated by the doctor who had admitted the patient to ICU. The endpoint of the study was in-hospital mortality.

### 2.3. Statistical Analysis

Distributions of quantitative variables were reported using means, standard deviations, medians and quartiles. Distributions of qualitative variables were described as the number and percentage of occurrence for each of their values. Qualitative variables were compared between groups using the chi-square test (with Yates’ correction for 2 × 2 tables). Fisher’s exact test was used in the case of low values in contingency tables. Quantitative variables were compared between two groups using the Mann–Whitney test. The impact of selected variables on dichotomous outcome was analysed using logistic regression. Odds ratios (ORs) with 95% confidence intervals were reported. The significance level for all statistical tests was set at 0.05. All computations were performed using R statistical software (v4.3.0.).

## 3. Results

### 3.1. Patient Characteristics by Sex

As a first step, we assessed differences between female and male patients. Female patients were significantly older than male patients (68.62 ± 14.77 vs. 62.7 ± 13.95) and were more likely to have an initial rhythm of asystole/PEA (Table 1).

### 3.2. Patient Characteristics by Presence or Absence of Obesity

No significant differences in the parameters analysed were found between female patients with obesity and non-obesity. Male patients with obesity were significantly older and had a significantly higher potassium level compared to non-obese group (Table 2).

### 3.3. Impact of BMI on Mortality

In the univariable logistic regression model, BMI was not a significant independent predictor of the odds of in-hospital death in either male or female patients (Table 3—MODEL 1).

In the multiple logistic regression (model 2), none of the traits analysed were a significant independent predictor of the odds of in-hospital death in female patients, whereas age was an independent predictor of the odds of in-hospital death in male patients (OR = 1.034)—each year of increase in age was associated with 3.4% higher odds of in-hospital death (Table 3—model 2).

In model 3 (adjusted for the remaining variables), none of the traits analysed were a significant independent predictor of the odds of in-hospital death in female patients, whereas an initial rhythm of VF/pVT was an independent predictor of the odds of in-hospital death in male patients (OR = 0.247)—male patients with an initial rhythm of VF/pVT had 75.3% lower odds of in-hospital death compared to male patients with an initial rhythm of asystole/PEA (Table 3—model 3). The final model included the most relevant clinical data obtained on admission to ICU, except for comorbidities. Information on the patients’ comorbidities was not available or not full available on their admission to hospital Table 3—model 3).

## 4. Discussion

The aim of the study was to assess the impact of BMI (calculated on admission to ICU) on in-hospital mortality in post-CA patients. We found no association between BMI and in-hospital mortality in either male or female patients. However, findings in this regard from studies by other authors are not conclusive.

In a study by Matinrazm et al., elevated BMI was associated with lower long-term mortality in OHCA and IHCA survivors, regardless of sex. Interestingly, elevated BMI remained a strong predictor of survival after adjustment for comorbidities and a number of other confounding factors (HR: 0.86 per increase by one BMI category). Underweight and normal-weight patients had the worst outcome [19]. According to the authors, this trend can be explained by the obesity paradox, i.e., the protective effects of obesity. A similar observation was made in a study by Gupta et al. based on data from more than 800,000 patients with IHCA, which found that obesity, recorded as a comorbidity (rather than BMI level), was associated with improved survival to hospital discharge. Similar results were found for patients with cardiovascular conditions and those with non-cardiovascular conditions as the primary diagnosis [22]. The obesity paradox is not a new discovery. It has been reported in a number of conditions [23,24,25,26], especially in CVD. Obesity is usually considered to be one of the main risk factors for CVD [13,27,28]. The obesity paradox remains controversial as its underlying cause has not yet been elucidated. One of the factors that may have an influence on the obesity paradox is age [29]. One meta-analysis analysing the relationship between BMI and mortality in patients with ACS in the context of the obesity paradox showed that patients with obesity were 1–10 years younger than normal-weight patients [30]. Patients with overweight/obesity are more likely to be tested earlier for CVD. This often results in earlier treatment, which in turn may affect prognosis [31,32]. In individuals whose lives are at risk, adipose tissue may provide nutritional reserves when metabolic demands increase greatly [33]. The protective association between obesity/overweight and clinical outcomes may also be due to the limitations of BMI. It does not consider the exact body composition, including body fat and muscle mass [34], fluid retention (e.g., in patients with heart failure) and does not distinguish between obesity phenotypes [35]. Sharma et al. noted that in many cases, the term “BMI paradox” would be more relevant [32]. A recent study has noted that the use of cardioprotective medications, smoking status and obesity duration may also play a significant role in the obesity paradox [36].

It should be stressed that excess adipose tissue can make cardiopulmonary resuscitation more difficult. The accumulation of large amounts of adipose tissue in the chest increases chest wall resistance during ventilation, which may necessitate the use of mechanical ventilation with higher positive pressure [37] and increase the risk of extubation failure (due to airway obstruction) [38].

A study by Lee et al. including patients resuscitated from OHCA did not find significant differences in survival outcomes between different BMI groups of patients. Only on univariate analysis were favourable neurologic outcomes more frequently observed in the obese group and less frequently observed in the underweight group. However, no association was found between the BMI classification and neurologic outcomes after adjustment for confounders [15]. Studies have confirmed an association between low BMI and poorer prognosis in CA survivors [39,40]. Some studies show that critically ill obese patients admitted to the ICU have a higher APACHE II (Acute Physiology and Chronic Health Evaluation) score than non-obese patients, regardless of whether obesity was classified by BMI ≥30 kg/m^2^ or waist-to-height ratio [41]. It is worth highlighting the fact that none of the commonly used disease severity scales (e.g., APACHE II) have been validated in obese patients and do not even take obesity into account [42]. This might be due to the fact that obesity can be associated with various comorbidities and physiological changes, which can affect the APACHE II score components. However, obesity can be associated with a variety of comorbidities and physiological changes [43] that can affect the components of the APACHE II score, for example.

In the present study, no association was found between being underweight and in-hospital mortality. However, the number of patients in our study with BMI < 18.5 was very small. The studies referred to above did not analyse differences between male and female patients.

Studies analysing sex differences among patients with ROSC focus on variables such as the initial rhythm of CA, witnessed status, episode location and emergency medical services response time [17,18,44,45,46]. Most of the studies investigating sex differences in the impact of BMI on mortality included patients with conditions that can lead to a CA, such as ACS [47], HF [48] and cardiogenic shock [49].

It is difficult to assess the nutritional status of post-CA patients on their admission to ICU due to dynamic changes in their condition and the priority of life-saving procedures. The present study did not find differences between male and female patients in the impact of BMI on the odds of in-hospital death. Our findings imply that further prospective research is necessary on factors related to sex differences and nutritional status and their impact on mortality among post-CA patients with ROSC.

### Study Limitations

The study had a number of limitations. One of them was that it only included a small number of patients. However, it should be noted that we focused on a very specific group of patients, namely those who had ROSC and could thus be admitted to ICU. Due to the condition of the patients, data on their comorbidities were uncertain. In the case of patients who never regained consciousness, data on comorbidities might have been provided by their families, who may not have had full information on the patients’ health. The patients’ body composition was not measured with bioelectrical impedance. Thus, information on the patients’ body fat levels was not available. As the patients’ data were anonymous, we could not assess their long-term survival.

## 5. Conclusions

BMI on admission to ICU is not a predictor of the odds of in-hospital death in either male or female post-SCA patients. Our study showed that an initial rhythm of VT/pVT was associated with 75% lower odds of in-hospital death in male post-SCA patients. The impact of BMI on in-hospital mortality in patients admitted to ICU due to an SCA requires further investigation.

## Figures and Tables

**Table 1:** Patient characteristics by sex.

Parameter		Female (N = 40)	Male (N = 89)	Total (N = 129)	*p*
LOHS (days)	Mean (SD)	8.72 (10.59)	8.28 (10.09)	8.42 (10.21)	0.955
	Median (quartiles)	5 (1–11.25)	4 (2–9)	5 (2–10)	
	Range	0–43	0–47	0–47	
In-hospital mortality	No	19 (47.50%)	38 (42.70%)	57 (44.19%)	0.752
	Yes	21 (52.50%)	51 (57.30%)	72 (55.81%)	
Age (years)	Mean (SD)	68.62 (14.77)	62.7 (13.95)	64.53 (14.41)	0.011 *
	Median (quartiles)	71.5 (61.75–78.25)	65 (56–73)	67 (57–74)	
	Range	28–88	26–88	26–88	
BMI (kg/m^2^)	Mean (SD)	26.93 (7.54)	27.64 (5.98)	27.42 (6.48)	0.374
	Median (quartiles)	25.71 (21.88–30)	26.3 (23.55–30)	26.23 (23.15–30)	
	Range	15.56–50.78	17.3–49.59	15.56–50.78	
BMI (kg/m^2^)	18.5–24.9	13 (32.50%)	28 (31.46%)	41 (31.78%)	*p* = 0.492
	<18.5	4 (10.00%)	3 (3.37%)	7 (5.43%)	
	25.0–29.9	14 (35.00%)	37 (41.57%)	51 (39.53%)	
	≥30	9 (22.50%)	21 (23.60%)	30 (23.26%)	
Initial rhythm of CA	Asystole/PEA	30 (75.00%)	41 (46.07%)	71 (55.04%)	0.004 *
	VF/pVT	10 (25.00%)	48 (53.93%)	58 (44.96%)	
CA episode location	OHCA	18 (45.00%)	47 (52.81%)	65 (50.39%)	0.529
	IHCA	22 (55.00%)	42 (47.19%)	64 (49.61%)	
ACS	No	40 (100.00%)	86 (96.63%)	126 (97.67%)	0.552
CS	No	40 (100.00%)	89 (100.00%)	129 (100.00%)	1
CKD	No	39 (97.50%)	89 (100.00%)	128 (99.22%)	0.31
HF	No	39 (97.50%)	88 (98.88%)	127 (98.45%)	0.526
DM	No	40 (100.00%)	89 (100.00%)	129 (100.00%)	1
HT	No	40 (100.00%)	89 (100.00%)	129 (100.00%)	1
Albumen (g/dL)	Mean (SD)	2.87 (0.69)	3.05 (0.77)	2.99 (0.75)	0.277
	Median (quartiles)	2.9 (2.5–3.4)	3.1 (2.55–3.65)	3 (2.5–3.52)	
	Range	1.3–3.9	1.4–4.9	1.3–4.9	
	Missing	3	14	17	
K (mmol/L)	Mean (SD)	4.8 (1.61)	4.66 (1.36)	4.7 (1.44)	0.797
	Median (quartiles)	4.52 (3.77–5.4)	4.3 (3.8–5.05)	4.38 (3.79–5.1)	
	Range	2.81–10	2.66–8.5	2.66–10	
	Missing	0	1	1	
Na (mmol/L)	Mean (SD)	138.68 (6.29)	138.65 (9.1)	138.66 (8.3)	0.918
	Median (quartiles)	138.5 (136–140.25)	138 (135–142)	138 (135–142)	
	Range	123–157	118–200	118–200	
	Missing	0	1	1	
hsCRP (mg/L)	Mean (SD)	59.04 (79.12)	40.47 (60.1)	46.17 (66.75)	0.192
	Median (quartiles)	22.31 (3.18–86.54)	11.7 (2.24–41.62)	13.68 (2.71–59.43)	
	Range	0.18–333.28	0.25–219.02	0.18–333.28	
	Missing	1	1	2	
PCT (ng/mL)	Mean (SD)	4.69 (9.67)	7.09 (31.38)	6.34 (26.55)	0.685
	Median (quartiles)	0.64 (0.1–4.64)	0.36 (0.16–2.34)	0.39 (0.12–3.03)	
	Range	0.01–55.28	0–207.37	0–207.37	
	Missing	0	1	1	

*p*—Qualitative variables: chi-square or Fisher’s exact test. Quantitative variables: Mann–Whitney test. * statistically significant (*p* < 0.05). Abbreviations: N, number of participants; LOHS, length of hospital stay; CA, cardiac arrest; BMI, body mass index; CKD, chronic kidney disease; HT, arterial hypertension; DM, diabetes mellitus; CS, cerebral stroke; ACS, acute coronary syndrome; HF, heart failure; DM, diabetes mellitus; K, potassium; Na, sodium; hsCRP, high-sensitivity *C*-reactive protein; PCT, procalcitonin; PEA, pulseless electrical activity; VF, ventricular fibrillation; pVT, pulseless ventricular tachycardia.

**Table 2:** Patient characteristics by presence or absence of obesity.

Parameter	Group	Female			Male		
		Non-Obese (N = 31)	Obese (N = 9)	*p*	Non-Obese (N = 68)	Obese (N = 21)	*p*
LOHS (days)	Mean (SD)	8.29 (10.39)	10.22 (11.77)	0.769	7.57 (9.5)	10.57 (11.78)	0.25
	Median (quartiles)	5 (1–9.5)	8 (1–12)		4 (2–8.25)	7 (2–14)	
	Range	0–43	0–33		0–47	0–41	
In-hospital mortality	Yes	16 (51.61%)	5 (55.56%)		39 (57.35%)	12 (57.14%)	1
Age (years)	Mean (SD)	68.61 (16.29)	68.67 (8.28)	0.582	60.65 (14.49)	69.33 (9.58)	0.008 *
	Median (quartiles)	71 (63.5–79)	72 (62–73)		63.5 (53–71)	71 (67–74)	
	Range	28–88	56–79		26–88	44–84	
Initial rhythm of CA	Asystole/PEA	25 (80.65%)	5 (55.56%)	0.19	31 (45.59%)	10 (47.62%)	1
	VF/pVF	6 (19.35%)	4 (44.44%)		37 (54.41%)	11 (52.38%)	
CA episode location	OHCA	13 (41.94%)	5 (55.56%)	0.705	36 (52.94%)	11 (52.38%)	1
	IHCA	18 (58.06%)	4 (44.44%)		32 (47.06%)	10 (47.62%)	
ACS	No	31 (100.00%)	9 (100.00%)	1	66 (97.06%)	20 (95.24%)	0.559
CS	No	31 (100.00%)	9 (100.00%)	1	68 (100.00%)	21 (100.00%)	1
CKD	No	30 (96.77%)	9 (100.00%)	1	68 (100.00%)	21 (100.00%)	1
HF	No	30 (96.77%)	9 (100.00%)	1	68 (100.00%)	20 (95.24%)	0.236
DM	No	31 (100.00%)	9 (100.00%)	1	68 (100.00%)	21 (100.00%)	1
HT	No	31 (100.00%)	9 (100.00%)	1	68 (100.00%)	21 (100.00%)	1
Albumen (g/dL)	Mean (SD)	2.85 (0.72)	2.96 (0.54)	0.969	3.04 (0.81)	3.06 (0.66)	0.864
	Median (quartiles)	2.95 (2.5–3.4)	2.9 (2.55–3.2)		3.05 (2.48–3.6)	3.1 (2.7–3.7)	
	Range	1.3–3.9	2.4–3.9		1.4–4.9	1.6–3.9	
	Missing	1	2		12	2	
K (mmol/L)	Mean (SD)	4.88 (1.75)	4.52 (1.03)	0.71	4.46 (1.3)	5.28 (1.38)	0.006 *
	Median (quartiles)	4.6 (3.77–5.52)	4.5 (3.78–4.7)		4.2 (3.62–4.88)	4.74 (4.3–5.83)	
	Range	2.81–10	3.34–6.72		2.66–8.27	3.4–8.5	
	Missing	0	0		1	0	
Na (mmol/L)	Mean (SD)	138.32 (5.9)	139.89 (7.74)	0.961	137.93 (6.36)	140.95 (14.82)	0.407
	Median (quartiles)	139 (136.5–140)	138 (136–141)		138 (135–142.5)	140 (138–142)	
	Range	123–157	131–157		119–159	118–200	
	Missing	0	0		1	0	
hsCRP (mg/L)	Mean (SD)	61.3 (83.65)	51.48 (65.43)	0.907	32.41 (51.53)	66.16 (77.71)	0.244
	Median (quartiles)	23.98 (2.96–87.75)	22.31 (5.51–69.5)		11.38 (2.63–34.75)	19.61 (2.14–133.36)	
	Range	0.18–333.28	0.46–199.32		0.25–217.45	0.85–219.02	
	Missing	1	0		1	0	
PCT (ng/mL)	Mean (SD)	3.25 (4.9)	9.66 (18.15)	0.871	8.54 (35.86)	2.45 (3.5)	0.363
	Median (quartiles)	0.64 (0.11–4.53)	0.23 (0.08–13.43)		0.34 (0.15–1.93)	1.42 (0.23–3.62)	
	Range	0.01–17.04	0.02–55.28		0–207.37	0.01–14.48	
	Missing	0	0		1	0	

* statistically significant (*p* < 0.05) *p*—Qualitative variables: chi-squared or Fisher’s exact test. Quantitative variables: Mann–Whitney test. Abbreviations: N, number of participants; LOHS, length of hospital stay; CA, cardiac arrest; BMI, body mass index; CKD, chronic kidney disease; HT, arterial hypertension; DM, diabetes mellitus; CS, cerebral stroke; ACS, acute coronary syndrome; HF, heart failure; DM, diabetes mellitus; K, potassium; Na, sodium; hsCRP, high-sensitivity *C*-reactive protein; PCT, procalcitonin; PEA, pulseless electrical activity; VF, ventricular fibrillation; pVT, pulseless ventricular tachycardia.

**Table 3:** Impact of BMI on mortality in male and female patients.

Model 1—Unadjusted						
	Trait		OR	95%CI		*p*
FEMALE	BMI	18.5–24.9	1.00	ref.		
		<18.5	0.857	0.091	8.075	0.893
		25.0–29.9	0.857	0.189	3.888	0.842
		≥30	1.071	0.194	5.913	0.937
MALE	BMI	18.5–24.9	1.000	ref.		
		<18.5	1.500	0.121	18.539	0.752
		25.0–29.9	0.984	0.365	2.653	0.975
		≥30	1.000	0.319	3.137	1.00
**Model 2—multiple logistic regression model, adjusted for age**						
	**Trait**		**OR**	**95%CI**		***p***
FEMALE	BMI	18.5–24.9	1	ref.		
		<18.5	0.856	0.09	8.119	0.892
		25.0–29.9	0.941	0.2	4.434	0.939
		≥30	1.117	0.2	6.239	0.9
	Age	(years)	0.987	0.944	1.032	0.558
MALE	BMI	18.5–24.9	1	ref.		
		<18.5	2.212	0.15	32.548	0.563
		25.0–29.9	1.016	0.367	2.811	0.976
		≥30	0.766	0.232	2.529	0.662
	Age	(years)	1.034	1	1.071	0.05 *
**Model 3—adjusted for the remaining variables**						
	**Trait**		**OR**	**95%CI**		***p***
FEMALE	BMI	18.5–24.9	1	ref.		
		<18.5	---	---	---	---
		25.0–29.9	0.673	0.117	3.874	0.658
		≥30	0.829	0.097	7.086	0.864
	Age	(years)	1.019	0.957	1.084	0.563
	CA Mechanism	Asystole/PEA	1	ref.		
		VF/pVT	1.419	0.237	8.489	0.701
	Albumen	(g/dL)	0.739	0.242	2.262	0.597
	hsCRP	(mg/L)	1.012	0.997	1.028	0.105
	PCT	(ng/mL)	1.131	0.926	1.381	0.232
MALE	BMI	18.5–24.9	1	ref.		
		<18.5	2.052	0.071	58.931	0.675
		25.0–29.9	1.323	0.347	5.044	0.682
		≥30	0.643	0.122	3.385	0.602
	Age	(years)	1.018	0.973	1.065	0.432
	CA Mechanism	Asystole/PEA	1	ref.		
		VF/pVT	0.247	0.072	0.856	0.027 *
	Albumen	(g/dL)	0.848	0.394	1.825	0.674
	hsCRP	(mg/L)	1.011	0.998	1.024	0.095
	PCT	(ng/mL)	1.163	0.863	1.567	0.321

* statistically significant (*p* < 0.05). Abbreviations: *p*, multiple logistic regression; OR, odds ratio; BMI, body mass index; CA, cardiac arrest; BMI, body mass index; hsCRP, high-sensitivity *C*-reactive protein; PCT, procalcitonin; PEA, pulseless electrical activity; VF, ventricular fibrillation; pVT, pulseless ventricular tachycardia.

## Data Availability

Not applicable.
